# The 2011 WPATH Standards of Care and Penile Reconstruction in Female-to-Male Transsexual Individuals

**DOI:** 10.1155/2012/581712

**Published:** 2012-05-14

**Authors:** Gennaro Selvaggi, Cecilia Dhejne, Mikael Landen, Anna Elander

**Affiliations:** ^1^Department of Plastic and Reconstructive Surgery, Sahlgrenska University Hospital, SE-41345 Gothenburg, Sweden; ^2^Division of Psychiatry, Department of Clinical Neuroscience, Karolinska University Hospital Huddinge, SE 14186 Stockholm, Sweden; ^3^Institute of Neuroscience and Physiology, Sahlgrenska University Hospital, Gothenburg, Sweden

## Abstract

The World Professional Association for Transgender Health (WPATH) currently publishes the Standards of Care (SOC), to provide clinical guidelines for health care of transsexual, transgender and gender non-conforming persons in order to maximize health and well-being by revealing gender dysphoria. An updated version (7th version, 2011) of the WPATH SOC is currently available. Differences between the 6th and the 7th versions of the SOC are shown; the SOC relevant to penile reconstruction in female-to-male (FtM) persons are emphasized, and we analyze how the 2011 WPATH SOC is influencing the daily practice of physicians involved in performing a penile reconstruction procedure for these patients. Depending by an individual's goals and expectations, the most appropriate surgical technique should be performed: the clinic performing penile reconstruction should be able to offer the whole range of techniques, such as: metoidioplasty, pedicle and free flaps phalloplasty procedures. The goals that physicians and health care institutions should achieve in the next years, in order to improve the care of female-to-male persons, consist in: informing in details the individuals applying for penile reconstruction about all the implications; referring specific individuals to centers capable to deliver a particular surgical technique; implementing the surgery with the most updated refinements.

## 1. Background

### 1.1. Gender Dysphoria

Gender dysphoria (GD) refers to discomfort or distress that is caused by a discrepancy between a person's gender identity and that person's sex assigned at birth (and the associated gender role and/or primary and secondary sex characteristics) [1, 2]. When the gender dysphoria reaches a significant level of distress, it can meet the criteria for a formal diagnosis: transsexualism in ICD 10 or gender identity disorder (GID) in DSM IV [3, 4]. The diagnosis is based on the person's description of incongruence between gender identity and phenotype, and the assessment of this by mental health professionals. There is currently a political movement against inclusion of transsexualism in diagnostic manuals in order not to view transsexualism as a psychiatric disorder, or as a diagnosis per se.

The delineation of conditions involving GD started in Germany in the 19th century [5], but it was Harry Benjamin's publication “The transsexual phenomenon” on the subject in 1966 that led to the widespread use of the term transsexualism [6]; the first diagnostic criteria were outlined in 1968. He also proposed a treatment protocol for transsexual persons who requested phenotype changes to be in harmony with the person's gender identity [7]; this protocol later developed into the standards of care.

During the last decades, there has been an evolution from dichotomy towards diversity thinking. Most persons with gender dysphoria who sought medical treatment used to want full gender reassignment, which consists of living in the desired gender role, hormonal and surgical treatment as well as legal gender reassignment; this was reflected in the term “gender change.” Today, the picture is more diverse, and not everyone who seeks medical help for this condition wants the full treatment in order to relieve his/her gender dysphoria. There has also been a movement to depathologization of the condition, emphasizing that not all persons with gender nonconforming behaviors have distress, or suffer, or even want any medical treatment.

Just five years ago, “Transsexual individuals” were defined as those “who seek medical assistance in changing their physical sex in order for internal self-perception and physical attributes to become congruent, thus increasing self-comfort and social fit” [8].

However, the rights not to be victims of discrimination and stigmatization have been emphasized in the last years, and a new terminology (see Table 1) has rapidly evolved in order to have more appropriate words for these phenomena, and for people to make them self-understood.

The most recent epidemiological study and review about transsexualism reports prevalence ranging from 1 : 11,900 to 1 : 45,000 for male-to-female persons (MtF) and 1 : 30,400 to 1 : 200,000 for female-to-male (FtM) persons [9], with an increasing number of patients seeking assistance in the recent years [10]. 

The etiology of transsexualism remains unknown. Probably, its etiology is both biological (“nature”) and dependent on external psychological influence (“nurture”).

Former theories on the pathogenesis of the disorder were based mainly on psychological perspectives, without supportive evidence-based data. By contrast, studies from the past two decades carried out by the Swaab group in Amsterdam, The Netherlands [11–13], advocate a neurological basis for gender identity with sexual morphological differentiation of the brain. These researchers have found that the volume of the central subdivision of the bed nucleus of the stria terminalis—a brain area that is essential for sexual behavior—is larger in men than in women, with similar findings in transsexual persons (MtF persons have the same size in this specific brain area as their female counterparts, and FtM persons have a same-sized region as their male counterparts) [11–14]. Sex atypical activation of the hypothalamus has also been demonstrated in MtF transsexuals exposed to two putative female pheromones [15]. However, there are still some conflicting observations. A recent study of gray and white matter fractions, hemispheric asymmetry and volumes of the hippocampus, thalamus, caudatem and putamen did not show a female pattern in MtF persons compared to heterosexual male and female control [16].

### 1.2. Treatment: Gender Reassignment

Gender reassignment (which is a comprehensive treatment aiming to alter the phenotype with hormonal therapy and/or surgery) has been demonstrated to be the best solution available for persons affected by gender dysphoria, in order to ease their condition, facilitating person's gender role and altering sex characteristics [18, 19]. Evaluation and treatment is a multidisciplinary task.

Diagnostic assessments are performed by mental health professionals and aim to assess the gender identity, gender dysphoria, the impact of stigma related, secure the diagnosis, to rule out other reasons for gender dysphoria, and to investigate and treat any concomitant medical condition, engage in psychotherapy, counsel in treatment options, and ascertain eligibility and readiness for endocrine and surgical treatments and recommendations to medical and surgical colleagues.

The treatment aims to make the body as congruent as possible with the gender identity. Besides cross-sex hormonal treatment and surgery, additional treatment as hair removal and vocal treatment for male-to-females is often applied.

Furthermore some persons benefit from psychotherapy and social support, also including support during the coming out process, and informing the surrounding family, friends, work, and schools.

Many gender dysphoric persons would like to have children, and all health professionals should discuss reproductive options (freezing of sperms or oocyte) before starting any treatment that could alter the fertility.

Regarding teenagers, there is a slightly different protocol of treatment, but its discussion is out of the scope of this paper.

### 1.3. Gender Reassignment Surgery

Gender reassignment surgery (GRS) is a complex process of surgical procedures including both genital and nongenital surgery, performed to alter the phenotypic expression of the biological sex in order of alleviate gender dysphoria.

Genital procedures performed for transsexualism, such as vaginoplasty, clitorolabioplasty, penectomy, and orchidectomy in male-to-female patients, and penile and scrotal reconstruction in female-to-male patients are under the name of sex reassignment surgery (SRS) [18]. Female-to-male transsexuals might also seek for a vaginectomy.

Non-genital procedures such breast enlargement, mastectomy, facial feminization surgery, voice surgery, and other masculinization and feminization procedures complete the picture [18].

A comparative study of international centers performing GRS has been previously published [21]. This survey reported on the standards and policies used in 1995 by 19 clinics located in Europe and North America. The aim of this study was to develop more uniform standards of care.

### 1.4. The World Professional Association for Transgender Health and the Standards of Care

The World Professional Association for Transgender Health (WPATH) publishes and reviews guidelines for persons with gender dysphoria, under the name of Standards of Care (SOC); the overall goal of the SOC is to provide clinical guidance for health professionals to assist transsexuals, transgender, and gender nonconforming people with safe and effective pathways to achieving lasting personal comfort with their gendered selves, in order to maximize their overall health, psychological well-being, and self-fulfillment [8, 17, 23].

The original SOC were published in 1979, and revised in 1980, 1981, 1990, 1998, 2001 (6th version) [20], and ultimately in 2011.

This 7th version (2011) of the WPATH SOC is currently available from the web [17].

The SOC are based on the best available science and expert professional consensus. The overall goal of the SOC is to provide clinical guidance for health professionals to assist transsexual, transgender, and gender non-conforming people with safe and effective pathways to achieving lasting personal comfort with their gendered selves, in order to maximize their overall health, psychological well-being, and self-fulfillment. This assistance may include primary care, gynecological and urological care, reproductive options, voice and communication therapy, mental health services (e.g., assessment, counseling, and psychotherapy), and hormonal and surgical therapy.

### 1.5. Differences between the 6th and the 7th Versions of the Standards of Care

The 7th version of the SOC represents a significant departure from previous versions. “Changes in this version are based upon significant cultural shifts, advances in clinical knowledge, and appreciation of the many health care issues that can arise for transsexual, transgender, and gender nonconforming people beyond hormone therapy and surgery” [17].

The SOC are intended to be flexible in order to meet the diverse health care needs of the entire group of transsexual, transgender, and gender nonconforming persons. While flexible, they offer standards to guide treatments to achieve optimal health care.

Similarly to previous versions of the SOC, current criteria are clinical guidelines, which can be modified by individual health professionals and programs particularly in case of the following conditions: patient's unique anatomic, social, or psychological situation; an experienced health professional's evolving method of handling a common situation; a research protocol; lack of resources in various parts of the world; or the need for specific harm reduction strategies. These departures should be recognized as such, explained to the patient, and documented through informed consent for both quality patient care and legal protection [17]. SOC promote this type of documentation, also for research purposes and health care progress.

SOC are intended for worldwide use; nevertheless, WPATH acknowledges that much of the recorded clinical experience and knowledge in this area is derived from north American and western European sources. Across nations and cultures, there are differences in all of the following: social attitudes towards transsexuals, transgender, and gender nonconforming people; constructions of gender roles and identities; language used to describe different gender identities; epidemiology of gender dysphoria; access to and cost of treatment; therapies offered; number and type of professionals who provide care; legal and policy issues related to this area of health care [24].

While the 6th version of the SOC does not report references, in the 7th version every statement is justified by references from papers published on peer-reviewed journals.

Table 2 points out the major differences between the 6th [20] and the 7th [17] versions of the SOC.

## 2. Standards of Care and Genital Surgery

While many transsexual, transgender, and gender nonconforming persons find comfort with their gender identity, role, and expression even without surgery, for many other individuals surgery is essential and medically necessary to alleviate their gender dysphoria [25]. For this group, relief from gender dysphoria cannot be achieved without modification of their primary and/or secondary sex characteristics to establish greater congruence with their gender identity. Moreover, surgery can help patients giving them more and better “passibility”: which means feeling more at ease in the presence of sex partners, in venues such as physicians' offices, swimming pools, or health clubs.

Follow-up studies have shown a beneficial effect of sex reassignment surgery on postoperative outcomes such as subjective well-being, cosmetics, and sexual function [26–29].

WPATH SOC suggest that genital surgery should not be carried out until “(i) persistent gender dysphoria is documented by a qualified mental health professional; (ii) that the patient has a capacity to make a fully informed consent for treatment; (iii) that the patient has reached the legal age of majority in a given country (if younger follow the SOC for children and adolescents); (iv) significant medical or mental health concerns are present, they must be well controlled; (v) 12 months of continued hormonal therapy according to the patients gender goals (unless there is contraindications or the patient is unwilling to); (vi) patients have lived continuously for at least 12 months in the gender role that is congruent with their gender identity” [17]. Also, the age threshold is a minimum criterion, and not a recommended age for intervention.

Two referrals from qualified mental health professionals who have independently assessed the patient are needed for genital surgery (i.e., hysterectomy/salpingo-oophorectomy, orchidectomy, and genital reconstructive surgeries). If the first referral is from the patient's psychotherapist, the second referral should be from a person who has only had an evaluative role with the patient. Two separate letters, or one letter signed by both (e.g., if practicing within the same clinic) may be sent.

While a mental health assessment is needed for referral to hormonal and surgical treatments, psychotherapy is recommended but not considered as a requirement.

The SOC do not recommend a minimum number of psychotherapy sessions prior to hormone therapy or surgery [17].

The person is referred from the general practitioner to a psychiatrist or another mental health professional with a special competence in GD, and eventually to an endocrinologist. After 1-2 years of “living in an identity congruent gender role” (named “real-life experience” in the 6th version of the SOC), consisting in cross-dressing full time and taking hormonal treatment, under the control of both the psychiatrist and the endocrinologist, and once the gender dysphoria has been confirmed and well documented, the transsexual person is referred for surgery. Usually, surgery is performed in one-to-three surgical interventions for male-to-female patients, and two to more than five for FtM patients, depending on the type of treatment chosen [8, 18].

After the gender reassignment process has been completed, transsexual individuals still need health assistance for life, to monitor the general health conditions (hormonal treatment is for life); they might eventually require corrective surgery or psychological assistance, even long after the gender reassignment [18].

The surgeon involved in the treatment of gender dysphoria has not got the exclusive role of a mere technician. Rather, surgeons should have insight into each patient's history and the rationale behind the referral to surgery. In order to achieve this, surgeons must have in depth discussions with transsexual individuals seeking surgery, as well as working as a team with other health professionals who have been actively involved in patients' clinical care [17]. Best practice is achieved when the surgeon practices as part of an interdisciplinary gender team. In the absence of this, the surgeon must be confident that both the referring mental health professional(s) and the physician who prescribes hormones are competent in the assessment and treatment of gender dysphoria, as the surgeon is relying heavily on their expertise.

At the point when the surgeon considers the criteria for surgery have been fulfilled, a preoperative surgical consultation can take place. During this consultation, the procedure and postoperative course should be extensively discussed with the patient. Surgeons are responsible for discussing all of the following with patients seeking surgical treatments for gender dysphoria: (1) the different surgical techniques available (with referral to colleagues who provide alternative options); (2) the advantages and disadvantages of each technique; (3) the limitations of a procedure to achieve “ideal” results; surgeons should provide a full range of before-and-after photographs of their own patients, including both successful and unsuccessful outcomes; (4) the inherent risks and possible complications of the various techniques; surgeons should inform patients of their own complication rates with each procedure.

These discussions are the core of the informed consent process; this is both an ethical and legal requirement for any surgical procedure. Surgeons must be sure that patients have realistic expectation of the outcomes, and that the achievable result would alleviate patients' gender dysphoria.

This information should preferably be provided both orally and in writing, in a language in which they are fluent, and can be complemented with graphic illustrations. Patients should receive this information well in advance and have enough time to review it carefully. The elements of informed consent should always be discussed face-to-face prior to the surgical intervention. Questions can then be answered and the patient can provide written informed consent. “Because these surgeries are irreversible, care should be taken to ensure that patients have sufficient time to absorb information fully before they are asked to provide informed consent. A minimum of 24 hours is suggested.”

Patients should work with their surgeon to develop an adequate aftercare plan for the surgery [17].

## 3. Techniques for Penile Reconstruction

Most recent reviews [18, 22] in penile reconstruction for female-to-male patients confirm the difficulty of this peculiar surgery, in terms of possible complications and limits of the final achievable outcomes, with surgery necessitating several steps and high number of revisions (see Table 3).

Furthermore, scientific progress in penile reconstruction is slow, with a lack of controlled studies, high loss to followup, and lack of validated assessment measures. Nevertheless, a few recent long-term followups have highlighted important issues that allow for a better patient selection (avoiding false expectancy from patients) [18, 22, 30] and confirm their improved quality of life and sexual health following penile reconstruction [31].

Genital surgical procedures for FtM persons may include hysterectomy, ovariectomy (salpingo-oophorectomy), vaginectomy, metoidioplasty, scrotoplasty, urethroplasty, placement of testicular prostheses, and phalloplasty. For persons without former abdominal surgery, the laparoscopic technique for hysterectomy and salpingo-oophorectomy is recommended to avoid a lower-abdominal scar. Vaginal access may be difficult as most transsexuals are nulliparous and have not often experienced penetrative intercourse. Currently, there are several different operative techniques for penile reconstruction. Choices for a specific technique may be restricted by anatomical and surgical considerations, and by the individual's financial considerations [17, 18]. If the patient's goal is a neophallus of good appearance, standing micturition, sexual sensation, and/or coital ability, patients should be clearly informed that surgery would require several separate stages, with technical difficulties, and high likelihood of additional operations [17, 18].

Phalloplasty, using a pedicled or a free vascularized flap, is a lengthy, multistage procedure with significant morbidity that includes frequent urinary complications (urinary tract stenoses and fistulas (can be as high as to 20% to 40%), unavoidable donor site scarring, and occasionally necrosis (partial or total) of the neophallus (1-2%) [18, 22, 30].

Even metoidioplasty (clitoris enlargement), which in theory is a one-stage procedure for construction of a microphallus, often requires more than one operation, and standing micturition cannot be guaranteed. Furthermore, metoidioplasty results in a micropenis, without the capacity for standing urination or sexual intercourse [18, 22].

Finally, erectile function is difficult to achieve. The radial forearm flap requires an inflatable penile prosthesis, with a considerable failure/revision rate. When a latissimus dorsi myocutaneous free flap is used, sexual intercourse is possible by contraction of the muscle, which stiffens, but shortens, the penis, with no need of an inflatable implant.

Flaps harvested with bone (e.g., fibula and osteocutaneous radial forearm flap) do not need stiffeners, but this flap type results in a permanent erection [18].

For these reasons, many FtM transsexuals never undergo genital surgery other than hysterectomy and salpingo-oophorectomy [32].

Several variations exist among the different gender units, even on the same surgical technique: vaginectomy and scrotoplasty constitute an integrated part of the radial forearm flap phalloplasty at the Ghent University Hospital, but these procedures might not be performed at all when the same radial forearm phalloplasty is performed at other centers [30].

Quality of surgical results has been demonstrated to be one of the best predictors of the overall outcome of sex reassignment [33].

## 4. Discussion

In this paper, we present differences between the 6th and the 7th versions of the SOC, and we intend to analyze how the 2011 WPATH SOC is influencing the daily practice of physicians involved in counseling and performing a penile reconstruction procedure in FtM individuals.

Furthermore, we raise questions related to the applicability of the 2011 WPATH SOC within different healthcare organizations.

Authors of this paper have regularly attended WPATH meetings (previously HIBIGDA meetings), but have not been involved in the preparation of the SOC.

### 4.1. Flexibility of the SOC

The SOC put a lot of emphasis on “flexibility,” mainly because of significant cultural shifts, advances in clinical knowledge, and appreciation of the many health care issues that might differ between individuals. Clinical departures from the SOC may be needed depending by patients and context circumstances [17].

Even though “flexibility” for obvious reasons is fundamental to the SOC, allowing physicians to tailor the treatment according to the patient's specific needs, ethical questions arise as to factors that may impact on flexibility (such as “lack of resources”); to which extent is a physician or an health system allowed to propose or refuse a surgical procedure simply based on economic issues such as lack of resources?

### 4.2. Ethical and Economic Considerations When Planning Penile Reconstruction

SOC present a paragraph on entitled “Reconstructive versus Aesthetic Surgery,” defining “Aesthetic or cosmetic surgery” what is mostly regarded as not medically necessary, and therefore typically paid for entirely by the patient. In contrast, “reconstructive procedures are considered medically necessary—with unquestionable therapeutic results—and thus paid for partially or entirely by national health systems or insurance companies.” The vast majority of professionals agree that genital surgery for gender dysphoric patient cannot be considered purely cosmetic [17].

We agree with this concept. In case of genital surgery for patients affected by gender dysphoria, the scope of the surgery is definitely medically necessary for some of the patients. For the World Health Organization (WHO), “health is a state of complete physical, mental, and social well-being and not merely the absence of disease or infirmity.” In gender reassignment, the aim is to restore health, intended as physical, mental, and social well-being to transsexuals individuals; in order to restore health, (genital) surgery is trying to restore function (i.e., urinating in a the desired male or female manner, practicing sexual intercourse, possibly achieving orgasm; (better) passibility in the preferred gender).

As a result of this way of thinking, successful surgical treatments (including the more expensive ones) should be provided by the national health systems and insurance companies.

Nevertheless, economic issues arise. It is not within the scope of the SOC, and not within the scope of this paper, to discuss and prioritize medical treatments.

Furthermore, no evidence is currently available to assess and list cost/effectiveness of each surgical option. Health economic studies in this field are needed.

### 4.3. Legal Considerations

SOC put emphasis on “informed consent” and “legal protection.” SOC come from North America and Western Europe: both these areas are currently facing issues concerning “legal protection,” to the extent that the perception of “informed consent,” “signed by the patient,” has the double purpose of “informing” the patient adequately and “protecting” the physician from legal point of view.

However, informed consent, as a literal matter, in the absence of fraud, is redundant.

This is the reason why medical actions within the Scandinavian countries are taken without the need of an informed consent signed by the patient, once information has been accurately given to the patient and documented in medical records.

### 4.4. Healthcare Organization and Penile Reconstruction in Transsexual Patients

Authors of this paper have personal experience with gender teams and penile reconstruction procedures offered in most of the European countries. Currently, it rarely happens that a single clinic is able to offer all the surgical techniques available for penile reconstruction. This is reflected in the scientific publications, which is clearly showing that a center for excellence in a metoidioplasty might have only minimal, if not any, experience with a free radial forearm phalloplasty. It is very likely that this lack of experience with one specific technique might be reflected in the preoperative consultation, while the transsexual person is planning his surgery, and this might give that person impartial information, and subsequently it might influence on the transsexual persons' decision.

A further problem is posed by the distribution of Gender Units in different countries: in UK, the only center performing penile reconstruction in based in London. This means that transsexual persons from all over UK need to fly to London to receive their surgery. If this surgery (e.g., free radial forearm flap) requires long recovery (sometimes longer than one month) and/or high risk for readmissions to the hospital, in order to deal with the complications, or simply post-op reviews at the clinic (which can also involve different specialties), that patient will find great discomfort in travelling long distances, possibly several times, with subsequent loss of earning and increased cost for the national health system.

Finally, if a (national) center is not able to offer a specific technique, as suggested by the SOC, the physician should refer that individual to another center, possibly even located in another country. Referring an individual to another country further complicates his life (possibly more paperwork needed, more travelling, different language, possibly longer waiting list, etc.). All this will influence on the patient's care and quality of life.

### 4.5. Transsexual Persons Who Are Refusing or Being Refused Surgery

It is reported in the literature [32] that some transsexual persons are simply refusing surgery [30]: no literature is reporting an updated statistic about this. The long-term outcome of transsexual patients operated in Sweden has been recently reported [34]; in the period 1973–2003, 324 persons with a diagnosis of transsexualism (both FtM and MtF) received a new legal gender. However, these 324 “operated transsexuals patients” were a subset of a total of 804 identified persons with a diagnosis of transsexualism that the author identified. Hence, 480 transsexual persons did not get a new legal gender. It is not known if this group received sex reassignment surgery outside Sweden or has not received, or did not want surgery at all.

More investigations are needed to understand why some transsexual persons prefer not to have sex reassignment surgery, and to analyze their quality of life, especially for those patients that wished to receive surgery. One explanation could be that they are concerned with their reproductive ability, but this has to be evaluated.

Similarly, another study is needed to evaluate the quality of life of patients that could not receive their first choice surgical technique, but accepted to go for their second or even third choice.

### 4.6. The Role of Gender Teams and Specialized Clinics

The SOC pose emphasis on the close working relationships with other health professionals who have been actively involved in the clinical care of the patients, such as surgeons, mental health professional(s), and if applicable the physician who prescribes hormones [17]. Monstrey et al. have reported the evidence that a close cooperation of the different specialties within the gender team is the key to success in treating transsexual patients [35].

This is not excluding isolated physicians from performing penile reconstruction; however, because of the complexity of the surgery, as well as the management of the patient, it is much easier to centralize the surgery in few centers, where expertise from different disciplines (endocrinology, psychiatrist, plastic surgeon, gynecology, and urologist) is easily accessible, rather than fragmenting the care of the transsexual patients among different clinics.

### 4.7. SOC: Information about Surgery

The 7th version of the SOC has got appendices about: (1) glossary, (2) overview of medical risks and hormone therapy, (3) summary of criteria for hormonal therapy and surgery, (4) evidence for clinical outcomes of therapeutic approaches, and (5) development process for the Standards of Care version 7.

Particularly, there is no table listing all the different surgical techniques available for penile reconstruction, with advantages and the disadvantages of each technique explained with details and percentages. On the opposite, there is a detailed table for risks associated with hormonal therapy. Similarly, within the text, there are 27 pages dedicated to hormonal therapy, and only 10 pages dedicated to surgery.

Our comment is not intended to remark on the SOC; but we believe that the mismatch between the large amount of information given within the SOC regarding hormonal therapy, and the paucity of information given regarding surgery, is simply the consequence of the wide experience, the scientific evidence, and finally the general consensus reached with the hormonal therapy, while surgery (penile reconstruction) is yet to have found the gold standard, a common consensus, or a worldwide preferred technique (by both surgeons and patients). As a consequence, currently there is no evidence and not even a suggested algorithm in guiding surgeons and patients in selecting the most appropriate penile reconstruction technique.

With new long-term followups and reviews have [18, 22, 31, 36] been published in the last few years, and others expected (regarding long-term followups of the most modern techniques as well as surgical refinements) from the scientific literature, SOC will soon need another update, which should put more emphasis on this surgery (penile reconstruction).

### 4.8. Timing between Consultation and Surgery

As understood from the SOC, an “ample” amount of time is required to review information about a treatment, but a minimum of 24-hour time period is suggested between face-to-face discussion of the informed consent with the surgeon, and the moment when patients are asked to provide inform consent.

SOC do not provide any exact information about timing between face-to-face discussion with the surgeon and surgery itself, and about the number of preoperative consultations that a patient should receive, before going ahead with the surgery. Nevertheless, the SOC are very clear about too large amount of information that the patient should receive (including advantages and disadvantages of each technique with limitations to achieve ideal results, full range of before-and-after photographs of surgeon's own patients including both successful and unsuccessful outcomes, and surgeon's own complication rates with each procedure), and the possibility for the surgeon to refer the transsexual person to a colleague performing a different technique.

For an operation such as penile reconstruction, where several options are available, results and surgical expectations can be very different, and complications are not uncommon, “ensuring that patients have a realistic expectation of outcomes” is fundamental, and the surgeon must be sure that the transsexual individual is taking the right decision.

In our view, the “minimum time” between face-to-face consultation and surgery should be extended: for elective procedures, within UK, most of the surgeons apply a 1-2 weeks minimum time before proceeding with surgery. Furthermore, in order for the surgeon to be sure that the patient has absorbed all the information, we would recommend a minimum of 2 face-to-face consultations with the surgeon, before proceeding with surgery.

The information provided by the SOC, in the way they are presented, might mislead the surgeons: more specifically, the surgeon might feel comfortable that providing internet information to the patient few weeks before, and having a face-to-face discussion with the patient about the informed consent just the day before the surgery, might suffice the criteria for operating the patient as early as the day after the first and only face-to-face discussion. We would disagree with this.

## 5. Conclusion

The World Professional Association for Transgender Health has currently published guidelines, named Standards of Care for persons affected by gender identity disorder.

In female-to-male genital surgery, the variety of techniques available for penile reconstruction demonstrates that the ideal technique has not yet been identified and, depending on the individual's request, different surgical approaches can be used.

Besides guidelines about diagnosis and hormonal treatment, the Standards of Care attempt to give guidelines about the approach to the transsexual persons referred for gender reassignment surgery.

The different surgical techniques available for penile reconstruction should all be explained to female-to-male transsexual persons. Depending on the individual's goals, the most appropriate surgical technique should be performed. Ideally, the clinic performing penile reconstruction should be able to offer the whole range of techniques, such as metoidioplasty, pedicle and free flaps phalloplasty procedures.

To date, it rarely happens that a single clinic is able to offer all these techniques, and that all the surgical refinements recently described would be incorporated in the chosen penile reconstruction technique.

Informing the transsexual persons in details, referring specific individuals to centers capable to deliver a particular surgical technique, and/or implementing the gender unit with the most updated surgical refinements are the goals that physicians and health care institutions should achieve in the next years, in order to improve the care of female-to-male transsexuals.

## Figures and Tables

**Table 1:** Definitions [17].

Gender nonconforming: adjective to describe individuals whose gender identity role or expression differs from what is normative for their assigned gender in a given culture or historical period.	
Gender dysphoria: distress that is caused by a discrepancy between a person's gender identity and that person's sex assigned at birth, and the gender role and/or primary and secondary sex characteristics.	
Transgender: adjective to describe a diverse group of individuals who cross or transcend culturally defined categories of gender. The gender identity of transgender people differs to varying degrees from the sex they were assigned at birth.	
Transsexual: adjective to describe individuals who seek to change or have changed their primary and/or secondary sex characteristics through feminizing or masculinizing medical intervention (hormones and/or surgery), typically accompanied by a permanent change in gender role. Some persons think this term is not ideal since it is objectifying people.	

**Table 2:** Differences between the 6th [20] and the 7th [17] versions of the Standards of Care.

SOC	6th version	7th version
Title	Standards of Care for gender identity disorders	Standards of Care for the Health of transsexual, transgender, and gender nonconforming people
Organization preparing the SOC	Harry Benjamin International Gender Dysphoria Association's Standards of Care for Gender Identity Disorders (HBIGDA)	World Professional Association for Transgender Health (WPATH), formerly HBIGDA
Date of publication	February 2001	Approved on September 14, 2011
Number of pages	22	120
References	Absent	Present
Based on	Clinical consensus	Clinical consensus and scientific references
View of relation between gender dysphoric persons and health personal	Health personal evaluate and treat a disorder	Health personal assist persons to better well-being and his/her harm reduction
Diagnosis or not	Diagnosis	Everyone with gender concerns does not have a diagnosis
View of diagnosis and evaluation and treatment	Five elements of clinical work: diagnostic assessment, psychotherapy or real-life experience, hormonal therapy, and surgical therapy	Different options of treatment
Tasks of the mental health professional	(1) to accurately diagnose the individual's gender disorder (2) to accurately diagnose and treat any psychiatric comorbidity (3) to counsel about the range of treatment options (4) to engage in psychotherapy (5) to ascertain eligibility and readiness for hormone and surgical therapy (6) to make formal recommendation to medical and surgical colleagues (7) to document their patient's relevant history in a letter of recommendation (8) to be a colleague in a team of professionals with interest in gender identity disorders (9) to educate family members, employers, and so forth about gender identity disorders (10) to be available for followup	(1) to assess the clients' gender dysphoria, the impact of stigma attached, and the support from the surrounding. The assessment may result in: no diagnosis, a diagnosis related to gender dysphoria, and/or in other diagnoses (the evaluation could also be done by a nonmental health professional if this person has appropriate training in assessing gender dysphoria) (2) to provide information regarding options for gender identity and expression, and possible medical treatment (3) to assess, diagnose, and discuss treatment options for coexisting mental health concerns (4) if applicable, assess eligibility, prepare, and refer for hormone therapy (5) if applicable, assess eligibility, prepare, and refer for surgery (6) to educate and advocate on behalf of the clients within their community and assist clients with making changes in identity documents (7) to provide information and referral for peer support
Psychotherapy prior to hormone treatment or surgery	Requirement if the patient did not experience three months of real life	Not a requirement
Real life	“Real-life experience” required for hormone and surgical treatment	“Living in an identity congruent gender role” required for genital surgery
One letter from the mental health professional required for instituting hormone therapy and breast surgery	The content of the letter is specified	The content of the letters is specified
Two letters from the mental health professional are generally required for genital surgery	The content of the letter is specified	The content of the letters is specified
Eligibility criteria for hormone therapy for adults	Age 18 years; demonstrable knowledge of what hormones medically can and cannot do and their social benefits and risks; either: (a) a documented real-life experience of at least three months prior to the administration of hormones; or (b) a period of psychotherapy of a duration specified by the mental health professional after the initial evaluation (usually a minimum of three months)	Persistent, well-documented gender dysphoria; capacity to make a fully informed decision and to consent for treatment; age of majority in a given country (if younger, follow specific SOC guidelines); if significant medical or mental health concerns are present, they must be reasonably well controlled
Readiness criteria for hormone therapy for adults	The patient has had further consolidation of gender identity during the real-life experience or psychotherapy; the patient has made some progresses in mastering other identified problems leading to improving or continuing stable mental health; the patient is likely to take hormones in a responsible manner	No differences between readiness and eligibility
Responsibilities of the hormone-prescribing physician	(1) hormones should not be prescribed before an adequate psychological and medical assessment (2) to review the likely effects and side effects of the hormone treatment	(1) perform an evaluation of the patient's physical transition goals, health history, physical examination, risk assessment, and relevant laboratory tests (2) discuss with patients the expected effects of feminizing/masculinizing medication and the possible adverse health effects including reduction in fertility (3) confirm that the patients have the capacity to understand the risks and benefits of treatment and are capable of making an informed decision about medical care (4) provide ongoing medical motoring, including regular physical and laboratory examination to monitor hormone effectiveness and side effects (5) communicate as needed with a patient's primary care provider, mental health professional, and surgeon (6) if needed, provide patients with a brief statement indicating that they are under medical supervision and care including feminizing/masculinizing hormone therapy
Can hormones be given to those who do not want surgery or a real-life experience?	Yes	Yes
Effects of hormone therapy on adults	Described	Larger amount of information is given, with detailed time course
Potential negative medical side effects	Described	Larger and detailed amount of information is given
The prescribing physician's responsibilities	Present	Present, more emphasized
Criteria for puberty suppressing hormones	Not present	Present
Hormones doses, misuse of hormones, and potential benefits of hormones	Present	Present, harm reduction is recommended
Clinical situation for hormonal therapy and risk assessment	Not present	Present
Information about hormones regimen	Limited	Larger amount of information given, all with references
Reproductive options	Limited	Larger amount of information given
Voice and communication therapy	Not present	Large amount of information given
Sex reassignment surgery	Proven to be effective, medically indicated	Proven to be effective, medically indicated
Ethical questions	Professional should feel comfortable about altering anatomically normal structures The resistance against performing on the ethical bases of “above all do not harm” should be respected	Professional should feel comfortable about altering anatomically normal structures The resistance against performing on the ethical bases of “above all do not harm” should be respected
HIV, HBV, and HCV	“Unethical” to deny treatment to HIV+, HBV+, HCV+, and so forth, patients	“Unethical” to deny treatment to HIV+, HBV+, HCV+, and so forth, patients
Surgeon's relationship with physician-prescribing hormones and mental health professional	They should work as a team. Surgeon should personally communicate with at least one of the mental health professionals to verify the authenticity of their letters	Close work relationship, working as a team
Informed consent	The medical records should contain written informed consent for the particular surgery to be performed	Larger amount of information is presented about the informed consent
Breast surgery	Minimal information given. No exact indication of timing between beginning of hormonal therapy, real-life experience and mastectomy. Mastectomy can be performed at the same time patients begin hormones. Augmentation mammoplasty may be performed 18 months after the beginning of the hormone treatment	Mastectomy to be performed preferably after ample time of living in the desired gender role, and after 1 year of testosterone treatment; however, hormone therapy does not constitute a prerequisite. It is suggested to perform augmentation mammoplasty after 1 year of hormone therapy
Criteria for hysterectomy and ovariectomy in FTM and orchidectomy in MTF	Not present	Persistent, well-documented gender dysphoria; capacity to make a fully informed decision and to consent for treatment; age of majority in a given country (if younger, follow specific SOC guidelines); if significant medical or mental health concerns are present, they must be reasonably well controlled; 12 continuous months of hormone therapy (unless the patient as a medical contraindication)
Genital surgery: eligibility criteria	Legal age of majority. Usually 12 months of continuous hormonal therapy for those without a medical contraindication 12 months of successful continuous full-time real-life experience Regular participation in psychotherapy, if required by the mental health professional Knowledge of cost, lengths of hospitalizations, likely complications, postsurgical rehabilitation requirements Awareness of different competent surgeons	Persistent, well-documented gender dysphoria; capacity to make a fully informed decision and to consent for treatment; age of majority in a given country (if younger, follow specific SOC guidelines); if significant medical or mental health concerns are present, they must be reasonably well controlled; 12 continuous months of hormone therapy (unless the patient as a medical contra-indication); 12 continuous months of living in a gender role that is congruent with their gender identity
		No difference between eligibility and readiness
Genital surgery: readiness criteria	Demonstrable progress in consolidating one's gender identity Demonstrable progress in dealing with work, family, and interpersonal issues resulting in a significantly better state of mental health; this implies satisfactory control of problems such as sociopathy, substance abuse, psychosis, and suicidal tendencies	
	No genital surgery possible without meeting the eligibility criteria	
Conditions under which surgery may occur	Written documentation that a comprehensive evaluation has occurred, and that the person has met the eligibility and readiness criteria Mental health professional, surgeon, and patient share responsibility of the decision to make irreversible change to the body	Provision of the information in writing, with illustrations, different techniques available, advantages, disadvantages, limits, risks, complications, informed consent, and so forth. Mental health professional and surgeon share responsibility of the decision to make irreversible change to the body
Requirements for the surgeon performing genital reconstruction	The surgeon should be urologist, gynecologist, plastic surgeon, or general surgeon. Board certified by a nationally known association	More emphasis on the fact that the gender surgeon/team should be able to offer several techniques More emphasis on the patient choice for the surgical technique
Other surgeries	Reduction thyroid chondroplasty, liposuction, rhinoplasty, facial bone reduction, face-lift, and blepharoplasty do not require letters of recommendation from mental health professionals. Voice modification surgery to be performed as last procedure, after that all other surgeries requiring general anesthesia with intubation are completed	Reduction thyroid chondroplasty, liposuction, rhinoplasty, facial bone reduction, face-lift, and blepharoplasty do not require letters of recommendation from mental health professionals
Competency of voice, communication specialists	Not present	Present
Information regarding phalloplasty	Patient should be clearly informed about limits of surgery, complications, stages of surgery, revision surgery Further technical developments of surgery are necessary Ideally, surgeons should be knowledgeable about more than one surgical technique for genital reconstruction so that they, in consultation with patients, can choose the ideal technique for each individual. Alternatively, if a surgeon is skilled in a single technique, referral to another skilled appropriate surgeon should be offered	Patient should be clearly informed about limits of surgery, complications, stages of surgery, revision surgery Pictures of successful and unsuccessful cases should be shown to the patients Ideally, surgeons should be knowledgeable about more than one surgical technique for genital reconstruction so that they, in consultation with patients, can choose the ideal technique for each individual. Alternatively, if a surgeon is skilled in a single technique, referral to another skilled appropriate surgeon should be offered
Urogenital care	Not present	Present
Posttransition followup	Followups recommended for both surgery, hormone treatments, and psychotherapy	Same as for 6th version
Life-long preventive and primary care	Not present	Present
Applicability of SOC to people with disorder of sex development	Not present	Present

**Table 3:** Techniques for female-to-male sex reassignment surgery [18, 22].

Surgical technique	Limitations	Benefits
Metoidioplasty (metaidoioplasty)	Short phallus Very rarely capable of sexual penetration Not always enable for voiding whilst standing Overall complication rate less than 20%	Easy technique Lower risk of complication Quick recovery time No donor-site morbidity

Phalloplasty		
Radial forearm flap	Urinary tract problems Multiple stages Stiffener required, or permanent erection if bone is used Donor-site morbidity Microsurgical skills required	Possible ability for sexual intercourse. Possibly, best cosmetic result? (overall complication rate up to 40%)
Anterolateral thigh flap	Possibly similar limitations to radial forearm flap No long-term followup available	Easier to hide the donor site disfigurement Usually harvested as a pedicle flap
Fibula flap	Possibly similar limitations to radial forearm flap Permanent erection No recent long-term follow-up available Microsurgical skills required	Easier to hide the donor site disfigurement
Latissimus dorsi flap	Urinary tract not reconstructed Erection function (based on muscle contraction) questionable Donor-site morbidity Sexual and tactile sensitivity not reported No long-term follow-up available Microsurgical skills required	No need of inflatable erection device
Suprapubic flap/groin flap	Cosmetic appearance unsatisfactory Donor-site morbidity? Urinary tract problem Fully or partially sensate? Stiffener or erection possible? Multiple stages If urethra is reconstructed, usually it is reconstructed in a different stage, and rarely reach the tip of the penis, but it often opens ventrally Groin flap requires a minimum of two stages	Easy technique
